# Mutual influences between the main olfactory and vomeronasal systems in development and evolution

**DOI:** 10.3389/fnana.2012.00050

**Published:** 2012-12-24

**Authors:** Rodrigo Suárez, Diego García-González, Fernando de Castro

**Affiliations:** ^1^Queensland Brain Institute, The University of Queensland, St LuciaBrisbane, QLD, Australia; ^2^Departamento de Biología, Facultad de Ciencias, Universidad de ChileSantiago, Chile; ^3^Grupo de Neurobiología del Desarrollo-GNDe, Hospital Nacional de Parapléjicos-SESCAMToledo, Spain

**Keywords:** odorant, pheromone, neuroethology, neurogenesis, axon guidance, cell migration, cerebral cortex

## Abstract

The sense of smell plays a crucial role in the sensory world of animals. Two chemosensory systems have been traditionally thought to play-independent roles in mammalian olfaction. According to this, the main olfactory system (MOS) specializes in the detection of environmental odorants, while the vomeronasal system (VNS) senses pheromones and semiochemicals produced by individuals of the same or different species. Although both systems differ in their anatomy and function, recent evidence suggests they act synergistically in the perception of scents. These interactions include similar responses to some ligands, overlap of telencephalic connections and mutual influences in the regulation of olfactory-guided behavior. In the present work, we propose the idea that the relationships between systems observed at the organismic level result from a constant interaction during development and reflects a common history of ecological adaptations in evolution. We review the literature to illustrate examples of developmental and evolutionary processes that evidence these interactions and propose that future research integrating both systems may shed new light on the mechanisms of olfaction.

## The hypothesis of dual olfaction

The ability to sense the chemical landscape has played an important role in animal evolution. The sensory systems involved in vertebrate chemoreception have specialized and diversified following a close relationship with the ecological conditions that animals face throughout both ontogeny and phylogeny. These two systems have been traditionally regarded as functional and anatomically independent, involving parallel processing of distinct sets of molecules, related to different behavioral contexts and involving distinct telencephalic connections [for specific review, see Ache and Young (2005)]. Consequently, this long held view has suggested that the processing of environmental odors occurs exclusively by the main olfactory system (MOS) and pheromones by the vomeronasal system (VNS). Therefore, the notion of “dual olfaction” refers to these seemingly independent paths of processing distinct stimuli resulting in distinct types of behaviors (Winans and Scalia, 1970; Scalia and Winans, 1975). However, in spite of anatomical and functional differences, recent findings strongly suggest that the MOS and VNS play synergistic roles in the regulation of a range of olfactory-guided behaviors, from foraging and defensive contexts to reproductive and social interactions, and show overlap in some of their central projections [for specific reviews, see Buck (2000); Dulac and Torello (2003); Ache and Young (2005); Baum (2012)].

We will discuss here the hypothesis that the synergistic actions of the MOS and VNS in vertebrates are not limited to olfactory perception and regulation of behavior, but they are also intimately related (1) during ontogeny, by sharing molecular and cellular processes, and (2) during phylogeny, by showing similar adaptations to changing ecological scenarios.

### General structure of the mammalian olfactory systems

**Figure 1:**
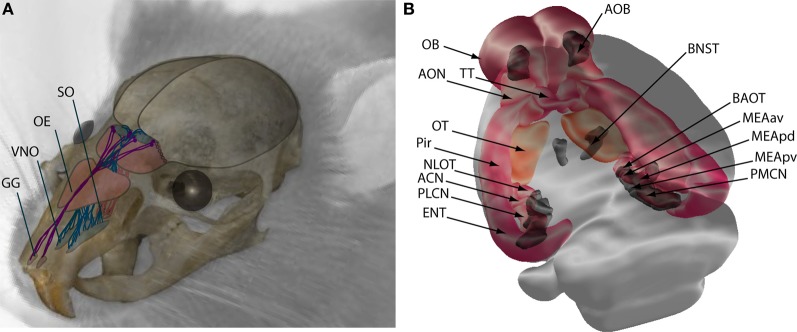
**Schematic representation of the main olfactory (MOS) and vomeronasal systems (VNS) in mice. (A)** Sensory neurons of the MOS are located in the nasal cavity at the olfactory epithelium (OE), Grüneberg ganglion (GG), and septal organ (SO), from where they send projections to the OB. Neurons of the VNS are located in the vomeronasal organ (VNO) and send axonal projections to the accessory olfactory bulb (AOB). **(B)** Central projections of the OB (pink and orange) terminate at the anterior olfactory nucleus (AON), tenia tecta (TT), olfactory tubercule (OT), piriform cortex (Pir), nucleus of the lateral olfactory tract (NLOT), anterior cortical nucleus (ACN) and posterolateral cortical nucleus (PLCN) of the amygdala, and entorhinal cortex (ENT). Central projections of the VNS are shown in dark gray, the accessory olfactory bulb (AOB) projects to the bed nucleus of the stria terminalis (BNST), the bed nucleus of the accessory olfactory tract (BAOT), the medial amygdala anteroventral (MEAav), posterodorsal (MEApd), posteroventral (MEApv), and posteromedial cortical nucleus (PMCN).

In the MOS, a group of olfactory sensory neurons (OSN) located at the roof of the nasal cavity [mainly at the olfactory epithelium (OE), plus two additional regions known as the Grüneberg ganglion and the septal organ] project to glomerular neuropil at the olfactory bulb [OB; Figure 1A; for a specific review, see Fleischer and Breer (2010)]. The OE consists of multiple laminar folds of neuroepithelium, where volatile molecules come into contact with the mucosa during either passive respiration or active sniffing. Each OSN extends multiple cilia into the mucous lining of the neuroepithelium, where only one olfactory receptor gene is expressed. Their activation triggers a cAMP cascade and the activation of cyclic nucleotide-gated and calcium-activated chloride channels [for a review, see Mombaerts (2004)]. There are millions of OSNs^1^ in the OE, each of them expresses just one of ~1000 olfactory receptor genes (it is the largest gene family within mammalian genomes; Buck and Axel, 1991), and those OSNs expressing the same receptor (~5000–10000 neurons) converge their axons to a single glomeruli at each half of the OB (Ressler et al., 1994; Vassar et al., 1994; Strotmann, 2001; Strotmann et al., 2004; Mombaerts, 2006; de Castro, 2009; Martínez-Marcos, 2009). Thus, each of the ~2000 glomeruli of the OB represents convergent projections of OSNs expressing the same receptor and is innervated by the apical dendrite of a single mitral/tufted cell (Mombaerts, 2006). This organization has been regarded as a “labeled line” of olfactory processing (Luo and Katz, 2004), as each mitral cell represents the activation of a single type of olfactory receptor and, after horizontal processing by periglomerular and granular interneurons, project to the telencephalic structures collectively known as olfactory cortex (OC; see below).

Conversely, the vomeronasal organ (VNO) is a close-ended tubular structure, located bilaterally at the base of the nasal septum, which opens to the mouth and/or nostrils, allowing influx of fluids containing pheromones by a vascular pumping mechanism performed by exploring animals (Meredith et al., 1980). Vomeronasal sensory neurons (VSNs) expose microvilli to the lumen of the VNO where they express a different subset of G-protein coupled receptors, either V1R or V2R, associated to either Gα_i2_ or Gα_o_ proteins, and project to rostral or caudal regions of the accessory olfactory bulb (AOB), respectively (Dulac and Axel, 1995; Berghard and Buck, 1996; Herrada and Dulac, 1997; Matsunami and Buck, 1997; Ryba and Tirindelli, 1997; Buck, 2000; Dulac and Wagner, 2006). Sensory transduction involves the activation of the IP3/DAG cascade and TRPC2 channels (Zufall et al., 2005). VRNs of apical or basal subdomains of the VNO express one receptor protein out of ~200 V1R or 80 V2R genes, respectively. However, in the AOB, each VSN projects to 6–30 glomeruli and each mitral cell innervates 3–9 glomeruli from neurons expressing the same or genetically related receptors (Belluscio et al., 1999; Rodríguez et al., 1999; Del Punta et al., 2002; Wagner et al., 2006; Larriva-Sahd, 2008). Therefore, it seems that sensory coding follows different rules between both chemosensory systems, with the VNS showing a high degree of synaptic integration from distinct receptors, possibly relating with a highly synthetic role in discerning qualitative aspects of pheromones (Holy et al., 2000; Luo et al., 2003; Ben-Shaul et al., 2010; Isogai et al., 2011).

The projections of the OB and AOB are remarkably different. The OB projects to the anterior olfactory nucleus, taenia tecta, olfactory tubercle, piriform cortex, anterior and posterolateral amygdaloid nuclei, and the lateral part of the entorhinal cortex. All of these projections form the medial and lateral olfactory tract (LOT), which also contains the equivalent efferents from the AOB (Figure 1B; Devor, 1976; Shipley and Adamek, 1984; Greer, 1991; Butler and Hodos, 2005; de Castro, 2009; Martínez-Marcos, 2009).

Recent studies have confirmed the existence of different projection patterns of OB efferents. Axons from mitral cells project widely to the OC, including the entire piriform cortex, while axons from tufted cells project to the more rostral structures, including the anterior portion of the piriform cortex (Haberly and Price, 1977; Nagayama et al., 2010; Fukunaga et al., 2012). These differences are related to different activation of mitral and tufted cells in response to OSN inputs and, consequently, diversify OB activity and processing signals on their way to the OC, ameliorating odorant discrimination (Fukunaga et al., 2012). The tertiary efferents from the OC structures can be summarized as follows: the piriform cortex projects to the endopiriform nucleus (connected, in turn, with the medial or vomeronasal amygdala, see below), the entorhinal cortex projects mainly to the hippocampus (forming the perforant path), and the olfactory amygdala project to the vomeronasal amygdaloid nuclei (Kang et al., 2009; Martínez-Marcos, 2009). In contrast, the AOB projects to medial striatal and subcortical amygdaloid nuclei, named the vomeronasal amygdala (Figure 1B; Winans and Scalia, 1970; Scalia and Winans, 1975; Devor, 1976; Shipley and Adamek, 1984; Price, 1986; Martínez-Marcos, 2009; Kang et al., 2011). The VNS seems to preserve some segregation between the areas of the AOB receiving input from VSNs expressing different vomeronasal receptors and their telencephalic projections (Mohedano-Moriano et al., 2007; Martínez-Marcos, 2009). The nuclei in receipt of AOB projections send tertiary projections exclusively to other structures of the VNS (including the AOB) and hypothalamic nuclei, with scarce fibers from the posteromedial amygdala making synapses on the hippocampal formation. Interestingly, there is a high degree of integration of centrifugal projections from nuclei in receipt of both OB and AOB projections (Mohedano-Moriano et al., 2007; 2008, 2012; Martínez-Marcos, 2009).

Despite the evidence supporting behavioral and anatomical differences between the MOS and the VNS, recent studies have shown an important overlap in the responses of each system to a range of ligands (odorants and pheromones), in the expression of receptors and other signaling components, in their telencephalic projections, and in their physiological and behavioral responses, prompting a review of the hypothesis of “dual olfaction” as it was originally raised [for specific reviews, see Brennan and Zufall (2006); Baum and Kelliher (2009)].

## Ontogenetic perspectives: the development of the main olfactory and vomeronasal systems

**Figure 2:**
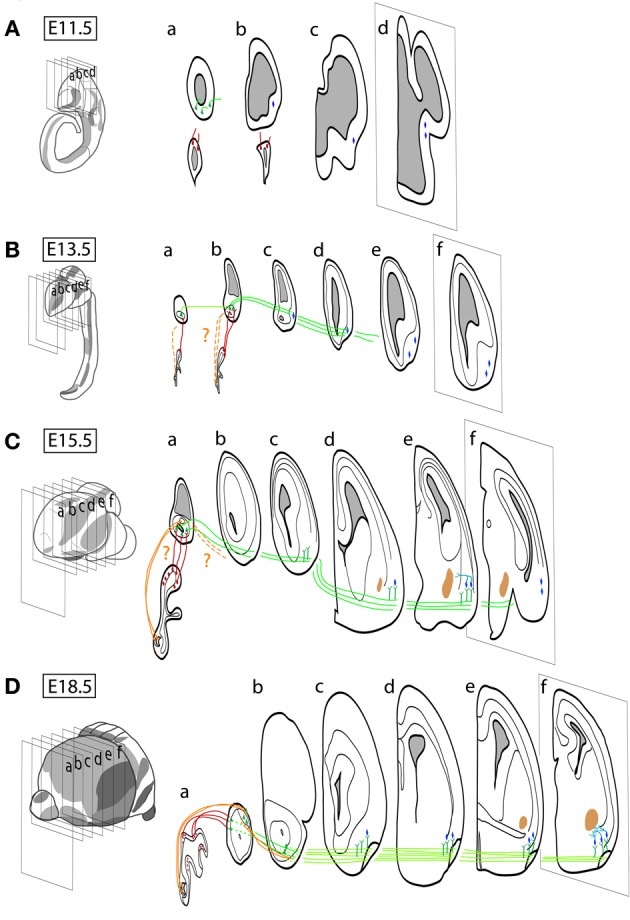
**Schematic representation of the spatio-temporal neurogenic pattern and its connections within the olfactory systems during embryonic development in the mouse. (A)** At approximately embryonic day 9.5 (E9.5), the olfactory placode starts to form with the first signs of neurogenesis in the presumptive OE, while the first neurons are generated by E11 at the OB and by E10 at the OC. **(B)** The first axons emerging from the olfactory placode are identified before E10.5, and they form clear olfactory and vomeronasal nerves between E11 and E12, which form synapses in the OB and AOB around E13.5 (dotted lines suggest the first axons from the VNO arriving into the AOB). **(A)** First LOT axons (AOB) are observed between E11.5 and E12 and **(B)** LOT covers the OC surface by E13.5. **(C)** Axon collaterals emerge from the LOT in gross amount E15.5, colonizing the OC structures. **(D)** OSN axons, diffusely innervating the OB at E13.5, reorganize topographically innervating protoglomeruli by E17.5 in a process which extends until postnatal stages (not illustrated). The maturation of synapses between OSN and OB neurons occurs by E17–E18, while AOB synapses mature by the end of the first postnatal week (not illustrated). The definitive OB and AOB layering depends on the arrival of the prospective interneurons generated in their neurogenic niche at the forebrain SVZ (see text for details).

Early in embryogenesis, the olfactory placode differentiates from the lateral surface ectoderm of the vertebrate head and gives rise to the sensory neurons reviewed in this work (OSNs and VSNs), as well as to gonadotropin-releasing hormone (GnRH) neurons (Schwanzel-Fukuda and Pfaff, 1989; Wray et al., 1989; Forni et al., 2011). All other olfactory and vomeronasal structures are telencephalic derivatives. A summary of the major events in development of these structures (i.e., neurogenesis-proliferation, cell migration and differentiation, axonal guidance and synaptogenesis) is illustrated in Figure 2, using the mouse time scale [for specific reviews, see López-Mascaraque and de Castro (2002, 2004); de Castro (2009)].

### Fate determination of olfactory and vomeronasal sensory neurons

As discussed above, the olfactory placode gives rise to three major neuron classes: OSNs, VSNs, and GnRH neurons. Despite growing evidence for the functional importance of these cell types, many aspects of their biology, such as the regulation of their cell fate and the molecular interactions at the OE as a neurogenic niche, remain poorly understood. These processes have become a subject of increasing interest as they may help unravel important questions regarding CNS regeneration, such as the production and functional integration of newborn neurons throughout ontogeny [for a specific review, see Schwob (2002)]. The functional identity of OSNs and their pattern of projections to the OB depend on the selective expression of a single olfactory receptor (Lomvardas et al., 2006). However, the overall identity of OSN precursors in subregions of the OE is affected by the graded expression of a set of molecules that act in a dose-dependent manner (Tucker et al., 2010). Although this graded pattern of expression reflects major differences between precursor cells in the lateral and medial portions of the OE, the existence of transition zones in the OE supports a combinatorial effect of factors modulating OSN fate choice (Tucker et al., 2010).

**Figure 3:**
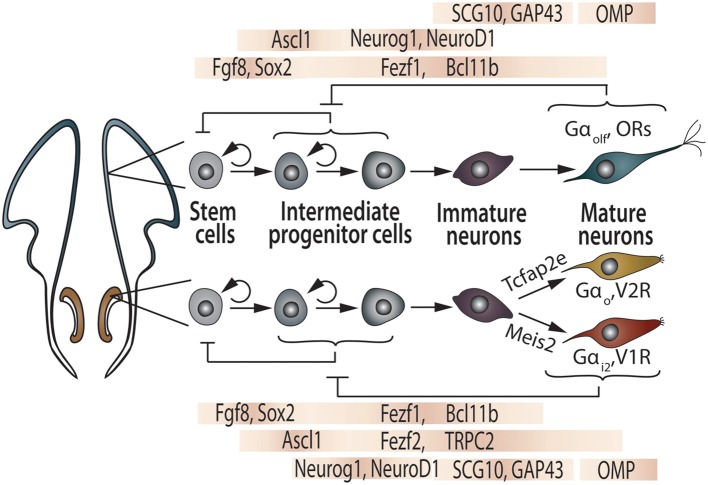
**Transcription factors controlling generation and maturation of OSN and VSN.** The OSN (top) and VSN (bottom) lineages are illustrated in parallel. Inhibitory feedback mechanisms are proposed to be acting on intermediate progenitor cells by mature neurons as well as regulation of stem cell pool by intermediate progenitors. See text and references therein for further explanations.

During early development of the olfactory pits, the expression of several molecules, such as the zinc-finger transcription factors Fezf1 and Fezf2, begins to differ between the presumptive vomeronasal and main olfactory regions. As early as E10.5, Fezf1 is expressed in both the OE and VNO, while Fezf2 expression is restricted to the VNO. By birth, Fezf1 becomes almost exclusively present in the OE, while Fezf2 retains its expression in the VNO (Figure 3; Eckler et al., 2011). Another zinc-finger transcription factor, BCl11b/Ctip2, plays an important role in the fate determination of VSNs into one of the two vomeronasal subtypes (Enomoto et al., 2011).

The development of OSNs and VSNs depends on complex interactions among proliferative and pro-differentiation transcription factors, sharing similar differentiation processes ruled by the neurogenic bHLH transcription factors Mash1, Ngn1, and NeuroD, which are sequentially expressed by neuronal progenitors, precursors, and differentiating neurons, respectively (Figure 3; Guillemot et al., 1993; Cau et al., 1997, 2002; Murray et al., 2003; Beites et al., 2005; Tucker et al., 2010; Enomoto et al., 2011; Packard et al., 2011; Suárez, 2011). These shared mechanisms may indicate that the genetic machinery involved in the maturation of functional chemosensory neurons has been conserved throughout evolution of both sensory systems. The frontal-nasal mesenchyme secretes essential inductive signals for the early development of the olfactory placode: the strong morphogen FGF-8 instructs the olfactory primordium to express high levels of *Meis1* (mainly at the lateral OE), as well as *Sox-2* and *Ascl1* genes (mainly at the medial OE). Altogether, these transcription factors regulate the differentiation from multipotent precursors to the three types of postmitotic neurons (Figure 3). Consequently, Meis1-expressing OE precursors have been recently implicated as the initial source of OSNs, VSNs, and GnRH neurons (Tucker et al., 2010).

### Zonal segregation and axonal pathfinding to OB and AOB glomeruli

**Figure 4:**
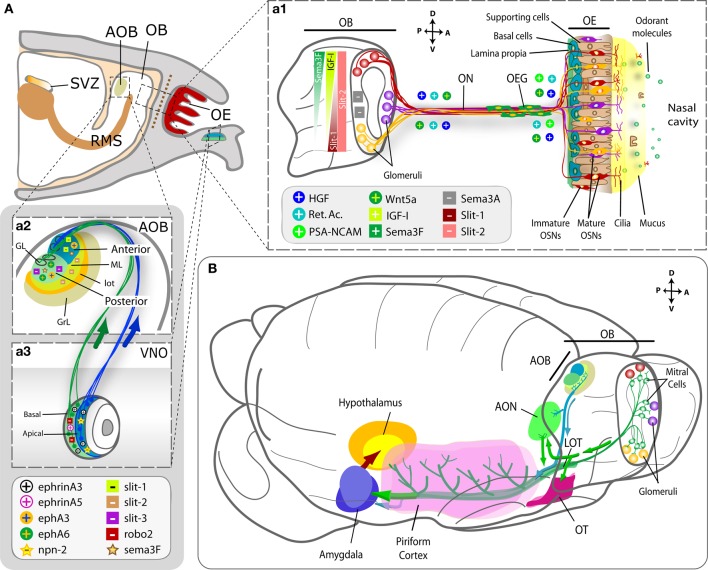
**Molecules involved in the formation of the olfactory and vomeronasal nerves and the LOT. (A)** Sagittal section of the rodent forebrain representing the olfactory structures. Panel **(a1)** represents the wiring between the OE and the OB, where each OSN expresses a single olfactory receptor gene and the axons from all cells expressing that particular receptor converge onto one or a few glomeruli (GL) in the OB. Molecules secreted from the OB form the olfactory nerve are represented as “+” when they attract/promote axon growth and as “−” when they repel/inhibit it. Spatial IGF-1, Sema 3A, Sema 3F, and Slit-1 gradients are crucial to address axons to their zonal targets within the OB. Panels **(a2** and **a3)** show in detail the VNO and the AOB structures and their connections, with layer-specific representation of the cues involved in the process. **(B)** Schematics showing the spatial relationship of the centripetal projections from the OB (in green) and AOB (in blue) to their recipient structures in the cortex. Abbreviations: AOB, accessory olfactory bulb; AON, anterior olfactory nucleus; GL, glomerular layer; GrL, granule layer; LOT, lateral olfactory tract; ML, mitral cell layer; OB, olfactory bulb; OE, olfactory epithelium; OEG, olfactory ensheathing glia; ON, olfactory nerve; OSN, olfactory sensory neuron; RMS, rostral migratory stream; SVZ, subventricular zone; OT, olfactory tubercle; VNO, vomeronasal organ.

Although the identification of olfactory receptors in the growth cones of the OSNs gave rise to speculations about their potential role in axon guidance, the relative contribution of these molecules versus the role of early sensory activity on olfactory targeting remains far for conclusive (Feinstein and Mombaerts, 2004; Priest and Puche, 2004; Mombaerts, 2006; Hovis et al., 2012). Most of these guidance processes relies on the orchestration of secreted chemoattractants orienting olfactory axons on their mesodermic way to the OB (such as HGF, retinoic acid, Wnt5a, with the collaboration of the adhesion molecule PSA-NCAM) as well as the intra bulbar gradients of both chemoattractants (IGF-1, Sema 3F) and chemorepellents (Sema 3A, Slit-1 and -2), which determine the pattern of OB glomeruli innervation (Figure 4Aa1; López-Mascaraque and de Castro, 2002; St John et al., 2002; Schwarting and Henion, 2008; de Castro, 2009; Imai, 2012).

On the contrary, relatively little is known about AOB glomerulus innervation. Depending on their basal-apical location, VSN axons express or not a battery of receptors (neuropilin-2, robo-1, ephA6) that affect their responses to the preliminary Sema 3F-due fasciculation and, once at the target, the production of Slit-1 (anterior part) or Slit-3/ephA6 (posterior part) within the AOB (Figures 4Aa2–a3).

Altogether, the available data reflect shared mechanisms between both systems in the organization of the first synaptic relay (secreted semaphorins and slits), as well as mechanisms that seem exclusive of the MOS (secreted morphogens—retinoic acid, Wnt, IGF-1, HGF-, the adhesion cue PSA-NCAM) or of the VNS (ephrins).

### Neurogenesis and cell migration in the olfactory forebrain

**Figure 5:**
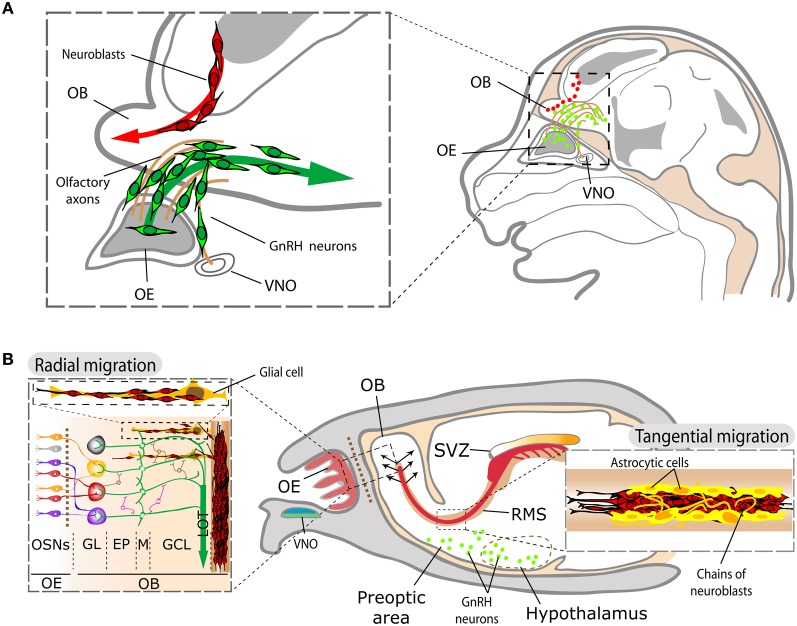
**Schematic representation of SVZ and GnRH neuroblast migration within the olfactory system. (A)** During development, neuroblasts originated in the lateral ganglionic eminences migrate toward the OB (red cells). GnRH-1 neuroblasts (green cells) generated in the OE follow the olfactory and vomeronasal axons on their way to the hypothalamus. **(B)** In the adult, the contingent of migrating SVZ neuroblasts (in red) forms the RMS toward the OB, whereas GnRH neurons (in green) are located in several areas comprising the preoptic area and the hypothalamus. The inset on the right illustrates the aspect of mature RMS, with the chains of migrating neuroblasts (in red) advancing among astrocytic channels (in yellow). Once they reach the OB, SVZ neuroblasts migrate radially to their final targets within the OB (left panel). Abbreviations: EP, external plexiform layer; GL, glomerular layer; GCL, granule layer; LOT, lateral olfactory tract; M, mitral cells; OB, olfactory bulb; OE, olfactory epithelium; OSN, olfactory sensory neuron; RMS, rostral migratory stream; SVZ, subventricular zone; VNO, vomeronasal organ.

One of the most striking characteristics of the olfactory system is that active physiological neurogenesis occurs throughout ontogeny. While in the OE new OSNs are generated to replace those dying, in the subventricular zone (SVZ) of the forebrain new interneuron precursors are generated which migrate and integrate in both the OB and AOB [Figure 5; for a specific review see Lledó et al. (2008)]. Both neurogenic processes start early in development (by E10.5 in a mouse-based chronology; Figures 2, 5) and the latter shows peaks just before (E17) and after birth (P2–P7), giving rise to its mature aspect a short time after that. By P15, the astrocytic channels allowing the migration of chains of newly generated neuroblasts are present (Petreanu and Álvarez-Buylla, 2002). Slit-2 forces neuroblasts to leave the SVZ neurogenic niche and (with the actions of motogenic and attractive anosmin-1, FGF-2, and HGF) migrate to form the rostral migratory stream (RMS), before reaching its mature aspect with astrocytic channels (Hu, 1999; Wu et al., 1999; Garzotto et al., 2008; García-González et al., 2010; Murcia-Belmonte et al., 2010). These active neurogenic processes make the OB an interesting model for the study of physiological plasticity in the adult brain. While fast-growing axons from newly generated OSNs project toward their respective OB glomeruli, a comparatively smaller contingent of newly generated interneurons (periglomerular and granule cells) differentiate and develop synaptic connections to integrate in functional micro-circuits, modulating firing properties of projection neurons (Belluzzi et al., 2003). Interestingly, the rate of arrival and maturation of new OB afferents from the OE is almost identical during postnatal development and in maturity, while the maturation of newly generated interneurons is remarkably slower in adults than in young animals (Carleton et al., 2003; Lemasson et al., 2005; Grubb et al., 2008; Lledó et al., 2008). The nature of the different dynamics of integration of new functional units from both neurogenic niches remains a challenge for researchers. However, the constant exposure of OSNs to the environment may imply high death rates, requiring a continuous renewal to assure reliable connections to the OB, while interneuron generation and replacement in the OB may relate to distinct olfactory memory capabilities/requirements throughout life and would be necessary to balance death of OB interneurons (Grubb et al., 2008; Mouret et al., 2009). This kind of olfactory *perpetuum mobile* converging in the OB perhaps makes it the CNS structure with the highest and most complex degree of synaptic plasticity, a particularity that would be taken as a reflection of what has been suggested for the developing OB (López-Mascaraque and de Castro, 2002, 2004).

### GnRH neurons: olfactory interactions and neuroendocrine control of behavior

Three different GnRH forms have been detected in vertebrates, each encoded by a different gene: GnRH-1 (the hypothalamic form, present in chromosome 8 in humans), GnRH-2 (the midbrain form in many mammals but not in mice or rats), and GnRH-3 (the nervous terminalis–telencephalic, primarily in teleosts), and only the first one appears to be associated to olfactory sensory pathways or KS pathogeny (Whitlock, 2005; Wray, 2010).

GnRH-1 neuroblasts display a second migratory process: once generated in the nasal pit, they follow olfactory and vomeronasal axons and enter the forebrain on the way to their final physiological location in the preoptic area and hypothalamus (Figure 4; Schwanzel-Fukuda and Pfaff, 1989; Wray et al., 1989). FGF8 is involved in the induction and differentiation of the mouse nasal placode and the loss of this morphogen results in the absence of VSN and GnRH-1 neurons (Kawauchi et al., 2005; Chung and Tsai, 2010). In agreement with this, out of all the different receptors involved in migration, FGFR1 is expressed by both SVZ and GnRH-1 neuroblasts to respond to FGF2- and/or anosmin-1 to reach the OB and the hypothalamus, respectively, during development (Cariboni et al., 2004; Gill et al., 2004; García-González et al., 2010). This capital role of FGFR1-signaling is also maintained in the SVZ neurogenic niche during adulthood (Figure 4). Indeed, the lack of migration of GnRH-1 neurons forms the basis of the hypogonadotropic hypogonadism observed in Kallmann syndrome, a genetic disorder from either FGFR1 and/or anosmin-1 gene disruption, which also results in anosmia (Dodé et al., 2003; Dodé and Hardelin, 2009).

The neuroendocrine GnRH-1 system is essential for vertebrate reproduction. In mammals, the number of GnRH-1-secreting neurons is low (~800 in the mouse), scattered from the OB to the hypothalamus, where the majority of them sends their axons to the medial eminence of the pituitary gland (Schwanzel-Fukuda and Pfaff, 1989; Wray et al., 1989). There, the pulsatile release of GnRH-1 to the portal capillary system controls secretion of gonadotropins from cells in the anterior pituitary and, consequently, the gonadal function (Gore, 2002; Foster et al., 2006).

As discussed in detail in the following section, mating behavior is dependent on chemosensory inputs from the MOS and VNS, whose central pathways contain fibers and cell bodies of GnRH-1 neurons. For instance, GnRH-1 can excite or inhibit neurons at the medial preoptic area (Pan et al., 1988), facilitate or suppress chemosensory responses in the amygdala (Westberry and Meredith, 2003); and stimulation of GnRH-1 receptors can cause short-term facilitation of sexual behavior (Dorsa and Smith, 1980; Sakuma and Pfaff, 1983). More recently, two independent tracer studies have shown that GnRH-1 neurons integrate to both MOS and VNS structures, primarily in the piriform cortex and the olfactory and vomeronasal amygdala (Boehm et al., 2005; Yoon et al., 2005). Consistent with this, some of these neurons in the vomeronasal (medial) amygdala are activated when female mice are exposed to the pheromone alpha-farnesene (produced by males in urine), inducing oestrus (Novotny, 2003; Boehm et al., 2005). Some neurons in the anterior cortical nucleus of the olfactory amygdala, mainly innervated by OB projection neurons (Dulac and Wagner, 2006) are activated by this pheromone, which suggests the involvement of the MOS in the response to pheromones, and raises the possibility that signals representing the same chemical, but originated separately in the OE and the VNO, may converge onto the same cortical neurons (Boehm et al., 2005). The presence of feedback loops between the hypothalamus and both the MOS and the VNS suggest that the neuroendocrine status of the animal may also modulate perception of these substances. Despite the relatively low number of GnRH-1 neurons, they have been reported to receive synaptic input from at least 10000 neurons in 26 different areas of the brain (including both MOS and VNS, but also regions involved in sexual behavior, arousal, reward, etc.) and send synaptic contacts to up to 50,000 neurons in 53 different brain areas involved in odor and pheromone processing, hunger, sexual behavior, defensive behavior, motility, etc. (Boehm et al., 2005). Altogether, these findings may suggest a mechanism in which GnRH-1 neurons integrate diverse information regarding the internal state of the animal and its external environment, modulating reproductive physiology and diverse functions to maximize reproductive success (Boehm et al., 2005).

### Early exposure to scents in the development of chemosensory systems

At birth, pups are exposed to a new and rich olfactory world that signal the position of their siblings, their mother, and the milk she provides. This chemical landscape is particularly relevant for altricial species, such as mice and rats, which are born with poorly developed visual and auditory senses and rely heavily on olfactory cues. Olfactory lesions in newborn pups lead to starvation by deficient nipple search and suckling behaviors, as at least more than half intact functional OMP^+^-OSNs are required to display these behaviors (Kawagishi et al., 2009). Suckling pups whose mothers are scented with artificial odorants develop a long-lasting preference for those scents (Hudson et al., 2002; Sevelinges et al., 2009), suggesting that olfactory imprinting is important for the development of adult olfactory preferences (Moriceau and Sullivan, 2005). Interestingly, the formation of early olfactory memories is associated with different emotional values with increasing relevance of the environmental context during the transition from full mother-dependence to weaning (Moriceau and Sullivan, 2006).

Early olfactory experience, possibly even before birth, may produce long-lasting effects in both the MOS and VNS (Garrosa et al., 1998; Hovis et al., 2012). Indeed, urine-derived pheromones applied to immature VSN *in vitro* induce proliferation and survival of progenitors, with the concurrent phosphorylation of *Erk*, *Ark*, and *Creb* genes (Xia et al., 2010). Similarly, exposure of young pups to mice urine of a different strain abolishes the development of adult preference to their own strain and promotes epigenetic alterations in vomeronasal receptor genes and other proteins involved in chemosignaling. This suggests that the early semiochemical landscape plays an instructive role in shaping the receptive profile of maturing VSNs (Broad and Keverne, 2012). Similarly, early postnatal chemoreception affects the formation of specific connections between OSN and OB glomeruli, olfactory learning capabilities, and olfactory receptor turnover (Zou et al., 2004; Kerr and Belluscio, 2006; Sawada et al., 2011). Moreover, OSN maturation coincides with the development of sensory selectivity during the first 2–3 weeks of age, and electrical activity at the OB is required for OSN plasticity and maintenance (Lee et al., 2011).

### Mutual interactions in the regulation of sexual behavior

As previously discussed, the existence of a functional independence between the olfactory and VNSs has been assumed for many years. However, it has recently been contradicted by new data providing evidence that the MOS is directly involved in pheromone-evoked behavior and endocrine responses, while the VNS regulates sex-specificity of the behavioral responses (Dulac and Kimchi, 2007). Whereas total bulbectomy abolishes both mating and aggressive behaviors, VNO surgical removal or chemical-induced OE ablation alone provokes more specific effects, suggesting the direct involvement of both the MOS and the VNS in the mediation of the neuroendocrine response to predator odors (Masini et al., 2010). More recently, selective genetic inactivation of OE has shown to be a useful tool to address this question. The genetic ablation of a cyclic nucleotide-gated channel (only expressed in most OSNs but not in VSNs) or type III adenylyl-cyclase activity (which blocks OSN signaling) in male mice leads to impaired sexual behavior and diminished aggressive responses toward intruders (Wong et al., 2000; Mandiyan et al., 2005; Yoon et al., 2005; Wang et al., 2006). Furthermore, in a different set of experiments, OB mitral cells in female mice were activated by a pheromone compound of male mouse urine (MTMT), suggesting the direct role of the MOS in pheromone detection (Lin et al., 2005). In summary, these results provide direct evidence that the MOS is required for the detection of some pheromones involved in sexual and aggressive behaviors.

Together with this, genetic ablation of the TRPC2 channel, essential for VNO-mediated pheromone signaling, causes several behavioral abnormalities in mice, consisting of indiscriminate courtship and mounting of males toward both males and females, and to the absence of aggression to male intruders (Leypold et al., 2002; Stowers et al., 2002). However, these male mice are able to display a normal mating behavior with females. These data demonstrate that VNS activity is not obligatory for launching mating behavior although it is necessary for sex discrimination and aggression between males.

## Evolutionary perspectives: phylogenetic interactions under changing ecological constraints

We have discussed that both chemosensory systems arise from similar pools of immature cells in spatially segregated regions of the developing nasal cavity, and that early developmental interactions regulate their differentiation into anatomically and functionally defined sensory systems with a partial overlap in their responses to ligands and central projections. In this section, we propose that the structure and function of these systems, as we observe today in extant mammals, are the result of mutual interactions under changing ecological scenarios, which can be traced back to their origins in the evolution of vertebrates.

### Evolution of vertebrate olfaction: underwater smelling

**Figure 6:**
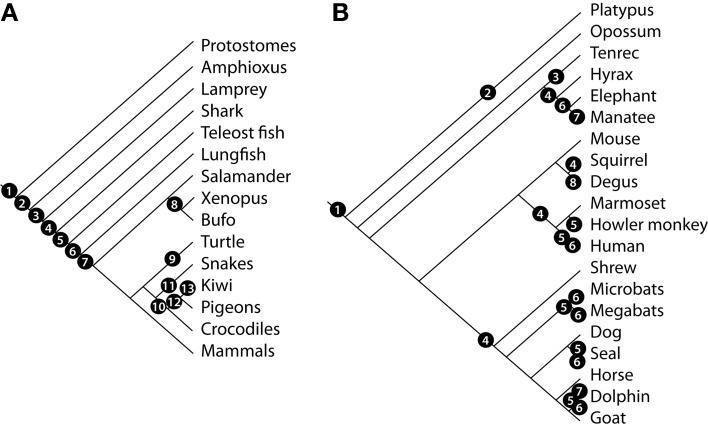
**Evolution of the vertebrate MOS and VNS.** Cladogram showing the phylogenetic relationships of vertebrate species and relevant events related to the evolution of the MOS and VNS **(A)** according to the following numbers: 1, presence of a primitive olfactory system; 2, evolution of the classical vertebrate olfactory receptor (OR) genes; 3, evolution of the olfactory projections and origin of V1R and TRPC2 genes; 4, origin of V2R genes; 5, expression of ORs, V2R, and V1R in the OE; 6, origin of a distinctive VNS (VNO-AOB-MeA projection); 7, segregation of vomeronasal pathways; shift in receptor ratios associated to land colonization; 8, differential expression of G-proteins in aquatic vs. terrestrial species; 9, reduction of OR gene repertoire in aquatic species; 10, loss of the VNS in Archosauria; 11, expansion of vomeronasal structures in lepidosaurs; 12, evolution of bird-specific OR genes; 13, expansion of OR gene repertoire in terrestrial/nocturnal birds. **(B)** Evolution of the mammalian MOS and VNS. Cladogram showing the phylogenetic relationships of mammalian species and relevant events related to the evolution of the MOS and VNS. 1, Two-pathway segregated VNS, AOB dorsal to the OB; 2, amplification of V1R genes; 3, non-exclusive segregation of AOB glomeruli expressing Gα_i2_ and Gα_o_ proteins; 4, loss of the V2R—Gα_o_ pathway; 5, pseudogenization of OR genes; 6, loss of the vomeronasal system; 7, loss of the OBs; 8, lateral AOB innervation and cell indentation between AOB subdomains.

Genomic analyses have shown the presence of intact olfactory receptor genes in amphioxus, suggesting that vertebrate-like olfaction was already present in early chordates, more than 500 million years ago (Churcher and Taylor, 2009; Grus and Zhang, 2009; Niimura, 2009), possibly having evolved from a group of genes present in the ancestors of protostomates and deuterostomates (Churcher and Taylor, 2011). Cyclostomes, such as hagfishes and lampreys, are the earliest extant chordates to show a distinctive olfactory system (Eisthen and Polese, 2007). Adult lampreys rely on olfaction for finding prey and sexual partners (Van Denbossche et al., 1997; Sorensen et al., 2005; Osório and Rétaux, 2008). However, it is not clear whether distinct neuronal structures are involved in distinct behaviors. The OSNs of lampreys project to a well-defined OB (Thornhill, 1967; Laframboise et al., 2007), and a subgroup of them, located at a structure termed accessory olfactory organ, terminate in medial domains of the OB (Ren et al., 2009). Interestingly, the lamprey genome has intact V1R and TRPC2 genes (Grus and Zhang, 2009), suggesting the evolution of alternative olfactory subsystems in early chordates (Figure 6A).

Both main olfactory and vomeronasal components are expressed in the OE of the teleost fishes in a structure known as olfactory rosette, through which water flows from the anterior and posterior nostrils. Distinct chemosensory neurons have been found to express ORs (Ngai et al., 1993a,b), V2Rs (Cao et al., 1998; Naito et al., 1998; Asano-Miyoshi et al., 2000; Pfister and Rodriguez, 2005), and V1Rs (Pfister and Rodriguez, 2005). Although fish do not present a compartimentalized VNO as in tetrapods, both ORs- and V1R-expressing neurons are spatially segregated in the OE (Hansen et al., 2004). Moreover, their projections to the OB terminate in non-overlapping glomerular domains (Sato et al., 2005), from where projecting neurons contacting each OE cell type project to the telencephalon in segregated bundles (Hamdani and Døving, 2007).

### Scents carried by the wind: the evolution of tetrapod olfaction

Air breathing is thought to have evolved in early bony fish (Sarcopterygii) with the ability of gulping air into primitive lungs, possibly as an adaptation to drops in atmospheric oxygen during the Middle and Late Devonian (Packard, 1974; Clack, 2007). Later increases in atmospheric levels of oxygen, concurrent with the colonization of land by plants, insects, and the evolution of shallow and fresh water habitats, may have further fostered aerial respiration in primitive lungfish and the diversification of tetrapods (Graham et al., 1995; Holland, 2006). Airborne substances may have acquired behavioral relevance after the fusion of the posterior nostrils into the mouth, forming the tetrapod-characteristic choana, allowing the flux of air into the olfactory cavity (Zhu and Ahlberg, 2004). This acquired ability may have further fostered the specialization of both the main olfactory and VNSs to distinct ecological contexts. In fact, lungfish (*Protopterus sp*.) possess the most ancient form of distinctive segregation between olfactory and vomeronasal pathways from the nose to the brain (González et al., 2010; Nakamuta et al., 2012; Northcutt and Rink, 2012). In accordance with this notion, Eisthen (1997) proposed that the evolution of the VNS was not associated to the evolution of terrestrial habits, as fully aquatic salamanders have a functional VNS. We advance this idea by proposing that the origin of vomeronasal components occurred in aquatic vertebrates before the split of lungfish and amphibians, and that the ability to take air into the nostrils may have allowed bimodal olfactory functions and promoted further specializations in both systems.

Amphibians display a rich diversity of behaviors involving the concerted action of the MOS and VNS (Eisthen, 1992, 1997; Park et al., 2004; Eisthen and Polese, 2007; Houck, 2009; Woodley, 2010). Evidence for a shift from fully aquatic to partially aerial olfaction came from the studies of Freitag et al. (1995), who described in *Xenopus laevis* three separate chemoreceptive chambers: the VNO, and the lateral (LD) and medial diverticulum (MD) of the OE. Both MOE diverticula are separated by a valve-like structure that allows the entrance of water to the LD or air to the MD for underwater and aerial olfaction, respectively. These OE subdomains show expression of ORs associated to waterborne (class I ORs) and airborne (class II ORs) chemoreception, respectively. Furthermore, while fully aquatic teleost fish have mostly class I ORs, coelacanths (Sarcopterygii) and amphibians have both OR classes (Freitag et al., 1998; Mezler et al., 2001; Niimura and Nei, 2005).

Thus far, the macroevolutionary transition from aquatic to aerial respiration in tetrapods involved a change in the repertoire of chemosensory elements, influencing the specialization of both olfactory and vomeronasal sensory systems. Evidence for this comes from genomic studies reporting a shift in the expression of ORs and VRs, with different affinities for waterborne and airborne molecules, during the evolution of terrestrial habits in vertebrates (Shi and Zhang, 2007).

The evolution of the amniotic sac further prompted the colonization of land by vertebrates, resulting in an increased independence to water resources for laying eggs. This allowed the diversification of chemosensory systems associated to life on land. Interestingly, species that returned to aquatic habits tend to lose olfactory functions. This has been reported in aquatic turtles, which show a reduction in functional OR genes as compared with terrestrial species (Vieyra, 2011). Similarly, sea snakes have less OR functional genes than terrestrial snakes; this genetic deterioration is more pronounced in viviparous than oviparous sea snakes, as laying eggs on land might rely more on a keen sense of smell than underwater delivery of alive newborns (Kishida and Hikida, 2010).

The interactions between the MOS and VNS are particularly evident in olfactory behaviors of diapsid reptiles. For example, the VNS of squamates (snakes and lizards) not only participates in intraspecific socio-sexual interactions, but also plays a fundamental role in finding prey (Cooper, 1996). Actively foraging snakes and lizards can follow the odorous trail of prey by performing tongue flicks, which deliver semiochemicals to the VNO through bilateral openings in the palate (Halpern and Kubie, 1980). In garter snakes (*Thamnophis sirtalis*), tongue-flicking behavior is often preceded by activation of the MOS, reflecting integration of both chemosensory modalities in predatory contexts (Halpern et al., 1997; Zuri and Halpern, 2003). The AOB projects to the vomeronasal amygdala, whose size reaches up to one third of total brain volume in some species, while the OB projects to the rostro-medial pallium, septum medialis, and lateral cortex (Halpern and Martinez-Marcos, 2003; Ubeda-Bañon et al., 2011).

Birds and their closest living relatives, crocodiles, lack a VNS. It has been postulated that the loss of vomeronasal structures dates back to the common ancestor of archosaurs (Senter, 2002). The complete lack of vomeronasal receptors in the chick genome (Shi and Zhang, 2007), further suggest an ancient loss of vomeronasal function. On the other hand, the relative size of the OBs in fossil and extant species suggests that olfaction has played an important role in the evolution of non-avian theropod dinosaurs and birds (Zelenitsky et al., 2011). Although the proportion of intact OR genes in birds is lower than in lizards, a recent expansion of a bird-specific OR gene family (Steiger et al., 2008), in addition to higher rates of OR diversification in nocturnal terrestrial birds (Steiger et al., 2009), suggest that olfaction in birds is more important than commonly thought (Figure 6A). In fact, olfaction in birds participates in recognition of gender (Balthazart and Taziaux, 2009) and individual identity of conspecifics (Coffin et al., 2011), as well as in foraging (Mardon et al., 2010) and navigation behavior (Gagliardo et al., 2011).

### Olfaction in mammals: sensory convergences to similar ecotypes

Fossil skull endocasts of basal mammaliforms from the Early Jurassic of China suggest that the evolution of the mammalian brain underwent a sequence of olfactory adaptations (Rowe et al., 2011). Similarly, olfaction has been proposed to have played an important role in the evolution of the isocortex of early mammals (Aboitiz et al., 2003).

The constant interactions between the MOS and the AOS during mammalian evolution are revealed by sharing similar patterns of diversification, between systems and between species, under similar ecological conditions. For example, the evolution of trichromatic vision in apes, and the perceptual changes associated to it, has been related to the sequential deterioration of vomeronasal components (Liman and Innan, 2003; Zhang and Webb, 2003). In some bats, the evolution of alternative communication systems or foraging strategies may explain the convergent deterioration of the vomeronasal genes (Zhao et al., 2011) Similarly, the OR gene repertoire has shown high levels of deterioration in New World and Old World primate species that convergently evolved trichromacy (Gilad et al., 2004). The independent acquisition of aquatic habits in several mammalian lineages has resulted in deteriorarion of OR genes (Kishida et al., 2007; Hayden et al., 2010) and VNS components in cetaceans, manatees, and some pinnipeds (Meisami and Bhatnagar, 1998). Accordingly, genomic analyses of receptor genes in mammals have shown that ecological adaptations affect the pattern of expression of components from both MOS and VNS. For example, OR gene repertoires cluster species acording to similar ecotypes (aquatic, semi-aquatic, terrestrial, flying) rather than by phylogenetic relatedness (Hayden et al., 2010). For instance, the amount of functional V1R genes is higher in nocturnal and nest-living species than in diurnal and open-living species, respectively (Wang et al., 2010; Young et al., 2010). In carnivores, ungulates and primates, the V2R gene family has degenerated (Young and Trask, 2007), possibly associated to independent origins of visually conspicuous sexual dimorphisms, as recently proposed as a possible explanation for the independent loss of the posterior AOB in squirrels and hyraxes (Suárez et al., 2011a; Figure 6B).

Interestingly, although in most mammals studied so far the vomeronasal nerve follows the medial line on its way to the AOB, in caviomorph rodents it follows a lateral course, regardless of the ecotype of species (Suárez and Mpodozis, 2009; Suárez et al., 2011b), reflecting that not all olfactory traits show correlation with life history but rather some traits can be shared within a phylogenetic group.

In summary, the similar directions of change observed in both MOS and VNS under different ecological adaptations reveal their constant mutual interactions throughout phylogeny. These interactions, together with their close relationship with the ecological contexts of species, may allow us to make predictions relating life history to olfaction (and vice versa) in less studied species and may provide valuable tools for conservation efforts.

## Concluding remarks

The recent findings of shared mechanisms between the MOS and VNS have challenged the notion of olfaction as a dichotomous process and instead suggest that many sensory and behavioral processes require the interaction between them. We have presented and discussed evidence supporting the hypothesis that these interactions are not only present during olfactory perception and generation of behavior at particular moments of the life of an individual, but rather they are constantly affecting the course of development and evolution.

This strong interaction is reflected in the molecular and cellular mechanisms controlling early development of both olfactory systems, to shared sensory activity and behavioral responses at postnatal stages and similar patterns of adaptation to environmental changes during evolution. The convergent patterns of ecological adaptations of both systems in species that acquire similar ecological niches may become instrumental for conservation efforts of endangered or poorly studied species through the effective prediction of lifestyle aspects based on olfactory structures and vice versa.

Furthermore, the study of the interactions between the MOS and VNS may open new routes of discovery of brain function. For example, the continuous production and integration of new neurons, both at the periphery (OE and VNO) and central structures (OB and AOB), may shed new light on the understanding of normal and pathological brain function, with possible applications in the design of therapeutic strategies for neural regeneration.

### Conflict of interest statement

The authors declare that the research was conducted in the absence of any commercial or financial relationships that could be construed as a potential conflict of interest.

## Footnotes

^1^The estimated number of OSNs is 5–10 million in rodents and humans and up to 50 million in rabbits.
